# Implications of cellobiohydrolase glycosylation for use in biomass conversion

**DOI:** 10.1186/1754-6834-1-10

**Published:** 2008-05-01

**Authors:** Tina Jeoh, William Michener, Michael E Himmel, Stephen R Decker, William S Adney

**Affiliations:** 1 Biological and Agricultural Engineering Department, University of California at Davis, Davis, California, USA; 2 National Bioenergy Center, National Renewable Energy Laboratory, 1617 Cole Blvd., Golden, CO 80401, USA; 3 Chemical and Biosciences Center, National Renewable Energy Laboratory 1617 Cole Blvd., Golden, CO 80401, USA

## Abstract

The cellulase producing ascomycete, *Trichoderma reesei *(*Hypocrea jecorina*), is known to secrete a range of enzymes important for ethanol production from lignocellulosic biomass. It is also widely used for the commercial scale production of industrial enzymes because of its ability to produce high titers of heterologous proteins. During the secretion process, a number of post-translational events can occur, however, that impact protein function and stability. Another ascomycete, *Aspergillus niger var. awamori*, is also known to produce large quantities of heterologous proteins for industry. In this study, *T. reesei *Cel7A, a cellobiohydrolase, was expressed in *A. niger var. awamori *and subjected to detailed biophysical characterization. The purified recombinant enzyme contains six times the amount of *N*-linked glycan than the enzyme purified from a commercial *T. reesei *enzyme preparation. The activities of the two enzyme forms were compared using bacterial (microcrystalline) and phosphoric acid swollen (amorphous) cellulose as substrates. This comparison suggested that the increased level of *N*-glycosylation of the recombinant Cel7A (rCel7A) resulted in reduced activity and increased non-productive binding on cellulose. When treated with the *N*-glycosidase PNGaseF, the molecular weight of the recombinant enzyme approached that of the commercial enzyme and the activity on cellulose was improved.

## Introduction

It is estimated that more than a billion tons of lignocellulosic plant biomass could be utilized each year to produce liquid biofuels in North America alone [1]. A major focus of research in the biofuels industry has been to address issues of enzyme production costs and performance. Developing low-cost enzymes remains a priority for both the Department of Energy's Office of the Biomass Program and for private industry. To achieve these goals, scientific advances in enzyme technologies, including enzyme production and performance, are required. Tactics proposed to solve the biomass conversion cost goals are diverse and include *in planta *enzyme expression, consolidated bioprocessing, enzymes engineered by rational design, enzymes improved by directed evolution, the development of hyper-producing fungal hosts, and many more. In each case, understanding those factors that impact the heterologous and/or hyper-expression of enzymes is critical for success.

Heterologous protein expression by fungi and yeast is complex and can be impacted by many post-translational events that take place during the secretion process. Fungal production and secretion of industrial enzymes can be highly productive and is the source for many industrial enzyme products. Yet, the fungal secretory pathway and the effects of post-translational events on protein function are poorly understood. Glycosylation is a common post-translational event that may have an effect on the properties of industrial enzymes. Glycosylation of glycosyl hydrolases, including cellulases, varies with the expression host and culture conditions. There is also evidence that different fungi used by industry, such as the *Trichoderma *and *Aspergillus*, glycosylate proteins differently. Therefore, because of the likelihood of critical impact on glycosyl hydrolase function and production, it is important to understand more about the chemical nature of the glycans decorating fungal enzymes, as well as their effect on cellulosic biomass conversion.

Glycosyl hydrolases represent a group of enzymes that hydrolyze the glycosidic bonds in carbohydrates [2]. Cellulases, a subgroup of glycosyl hydrolases, are specific for cleaving the β(1→4) bonds of cellulose, making them key enzymes for the economic production of cellulosic ethanol from ligncellulosic materials. To achieve complete hydrolysis of structured cellulose in biomass requires a synergistic mix of enzymes that include exoglucanase, endoglucanase, and cellobiase activities. The cost of these enzymes has been identified as a key economic barrier to the deployment of liquid fuels from lignocellulosics [3]. Long term strategies for bioethanol cost reduction include engineering noncellulolytic ethanologens to produce cellulases or engineering cellulase producers to become ethanologenic. Nearer term strategies currently rely on the over-expression of cellulase enzymes in heterologous production hosts. For both examples, consideration must be given to the co- and post-translational variations of heterologous proteins which may impact structure and function [4]. Protein glycosylation has been demonstrated to impact the function, structural framework, and stability of proteins [5]. A major challenge, therefore, for improvements in commercial cellulase production is to understand how protein glycosylation is impacted by expression from different hosts and specifically how glycosylation affects the activity of cellobiohydrolases. For example, glycosylation must be considered for the expression and production of cellobiohydrolases *in planta*, or for the development of advanced biocatalysts for consolidated bioprocessing. The significance of this problem is illustrated by considering the expression of *T. reesei *Cel7A in yeast. Expression of Cel7A in *Pichia pastoris*, for example, resulted in over-glycosylation of the enzyme with compromised activity [6,7].

Recent reports describe the successful co-production of an endoglucanase and β-D-glucosidase in *Saccharomyces cerevisiae*, as well as the production of ethanol from the hydrolysis of phosphoric acid swollen cellulose [8]. However, the expression of *T. reesei *Cel7A in *S. cerevisiae *has been less successful and generally results in the heterologous enzyme preparation having high and polydisperse molecular weights (MW) due to hyperglycosylation [9]. The apparent MW of the heterologous protein prior to secretion (intracellular) or after enzymatic deglycosylation of the purified enzyme was more monodisperse and closer to the native [9].

*T. reesei *produces a suite of cellulolytic enzymes with distinctly different activities, including endocellulases, exocellulases, and β-glucosidases which synergistically depolymerize cellulose [10,11]. Glycosyl hydrolases are classified into families based on amino acid sequence and folding similarities [2]. Family 7 consists of both endoglucanases and exoglucanases and is exclusively derived from eukaryotes. Cellobiohydrolases within the family are recognized as being the most important single enzyme component for the cellulose conversion industry. Of these, Cel7A from the industrial fungus *T. reesei*, is the most studied. Cel7A is produced abundantly by *Trichoderma*, typically making up to 60% of the total secreted protein [12]. This enzyme hydrolyzes cellobiose units from the reducing-end of a cellulose chain and is considered to be a processive enzyme [13]. As such, considerable attention has been paid to the study of *T. reesei *Cel7A [14-16] with the ultimate goal of improving its performance [17].

Most enzymes secreted by *T. reesei*, including Cel7A, are glycoproteins with both *O*- and *N*-linked glycosylation sites [18-23]. Cel7A consists of a catalytic domain (CD) and a cellulose binding module (CBM) joined by a highly *O*-glycosylated linker peptide [20]. The catalytic domain has four *N*-linked motifs, three of which are glycosylated [21]. The type and extent of glycan on the CD is influenced by both the strain [21] and culture conditions [21]. Fermentation pH is a dominant determinant for the final configuration of the glycans by influencing activities of co-secreted glycanases, such as endoglycosidase H (Endo H) [18], α-mannosidases [18,24,25], and α-glucosidases that modify the glycan structures in the medium [18].

Various expression systems, including *E. coli*, *Spodoptera frugiperda *(insect cells), *Pichia pastoris*, *Saccharomyces cerevisiae*, and *Aspergillus niger var. awamori *have been used for the heterologous expression of the *cel7a *gene [26]. Successful expression of functional protein has been limited due to stringent co- and post-translational requirements, including formation of disulfide bridges and native-like glycosylation. Of these hosts, *A. niger var. awamori *and insect cells produce functional enzyme but show greater extents of glycosylation compared to native enzyme [26]. The molecular mass of the recombinant Cel7A (rCel7A) was higher than the native protein and the reported data show slight differences in the activities of the two enzymes on pretreated yellow poplar [26]. *A. niger var. awamori *expressed enzyme had equivalent thermal stability to *T. reesei *Cel7A when tested by differential scanning calorimetry (DSC) [26].

In this study, we examine the effects of heterologous expression of Cel7A from *T. reesei *on glycosylation and activity. Understanding the heterologous expression of cellobiohydrolases and cellulases in general is important in the future development of fuels from cellulosic biomass.

## Materials and methods

### Substrate preparation

#### Bacterial Cellulose (BC)

BC was produced in static cultures of *Gluconacetobacter xylinus *sbsp. sucrofermentans (ATCC 700178) in Hestrin Schramm medium [27] with 1% (v/v) ethanol [28]. Inocula were prepared by growing frozen *G. xylinus *culture in 50 mL of the same medium (HS + 1% ethanol) at 26°C for 3 d under static conditions. At the end of the three days, the culture flask was shaken vigorously to dislodge the cells from the pellicle. The cells were pelleted by centrifugation and used to inoculate 75 mL media in 750 mL rectangular tissue culture flasks. Production cultures were incubated at 26°C for 5–7 d without agitation. At the end of the production period, the cells were re-pelleted and used in fresh media for growing subsequent batches of BC. The BC pellicles were washed as described by Helbert and co-workers [29] with the following modifications: 1) following neutralization from the alkali wash, the cellulose pellicles were incubated in a 0.3% bleach solution (in 4 mM sodium acetate buffer) for 2 h at 70°C, and 2) the pellicles were rinsed three times with distilled water to remove the bleach solution and subsequently resuspended in 5 mM sodium acetate buffer with 0.04% sodium azide and homogenized in a food processor. A final concentration of 1.9 mg/mL (standard deviation of 0.12 mg/mL) was determined from triplicate oven dry weights of 3 mL suspensions. The stock BC suspension was stored at 4°C.

#### Phosphoric acid swollen cellulose (PASC)

PASC was prepared from Sigmacell 50 (Sigma-Aldrich Co., St. Louis, MO). A cellulose slurry of 30 g Sigmacell in water was slowly added to 2.5 L of vigorously stirred, concentrated phosphoric acid (≥ 85 wt. % in water) at 4°C. The suspension was stirred at 4°C for 1 h before precipitating in 10–15 L of water. The precipitated cellulose was washed extensively with water until the final pH equilibrated at pH 5. The PASC was autoclaved and stored at room temperature. Final PASC concentration was 8.39 (± 0.03) mg/mL, as determined by triplicate oven-dried measurements of 3 mL suspensions.

### Enzyme Expression and Purification

#### Trichoderma reesei Cel7A

Cel7A enzyme was purified from Spezyme CP (Genencor International). The cellulase preparation was dialyzed extensively into 10 mM Bis-Tris buffer, pH 6.0 and loaded onto a HiPrep 16/10 DEAE Sepharose FF column anion exchange column. After extensive washing with buffer, bound proteins were eluted using a linear, 0 to 1.0 M NaCl gradient in 10 mM Bis-Tris buffer, pH 6.0. The collected fractions were analyzed by SDS-PAGE, using previously purified *T. reesei *Cel7A as a standard to identify fractions containing Cel7A. These fractions were pooled, desalted and loaded onto a 6 mL ResourceQ anion exchange column (GE Healthcare Bio-Sciences). The unbound fraction was washed out and the bound fraction was eluted with a linear gradient of 0 to 1.0 M NaCl in 10 mM Bis-Tris buffer, pH 6.0. The presence of Cel7A in the eluted fractions was determined by assaying for activity on *p*-nitrophenyl β-D-lactobioside (pNPL). Fractions that were active for pNPL activity were pooled, spiked with 1 mM gluconolactone and loaded onto a column packed with *p*-aminophenyl β-D-cellobioside (pAPC) affinity matrix [30]. The bound fraction was eluted with 10 mM cellobiose in 10 mM Bis-Tris buffer, pH 6.0. The eluted fractions that tested positive for pNPL activity were pooled, concentrated and run through a Superose 12 size exclusion column (GE Healthcare Bio-Sciences Corp., Piscataway, NJ) with 20 mM sodium acetate buffer, 100 mM NaCl at pH 5.0 as the running buffer. A single band corresponding to the MW of the *T. reesei *Cel7A standard on SDS-PAGE verified the purity of the eluted Cel7A.

#### Cloning and Heterologous Expression/Protein purification

rCel7A was produced in *A. niger var. awamori *and purified as described previously by Adney et al. [26].

#### Cel7A Catalytic Domains (CD)

Catalytic domains (CD) of Cel7A and rCel7A were obtained by proteolytic cleavage of the linker peptide with papain [31]. A ratio of 30:1 (w/w) of papain from *Carica papaya*, immobilized on Eupergit^®^C (76221 Sigma-Aldrich Co., St. Louis, MO) to Cel7A was used in 5 mM sodium acetate buffer, pH 5.0, and incubated overnight at room temperature. The digested samples were separated by size exclusion chromatography on a Superdex 200 16/60 column (Amersham Biosciences, Piscataway, NJ) in 20 mM sodium acetate, 100 mM sodium chloride, pH 5.0 at 0.5 mL/min. Fractions containing the CDs were pooled and concentrated in a 10,000 MWCO Amicon Ultra-4 centrifugal filter unit (Millipore Corp., Billerica, MA).

### PNGaseF Deglycosylation

**Figure 1:**
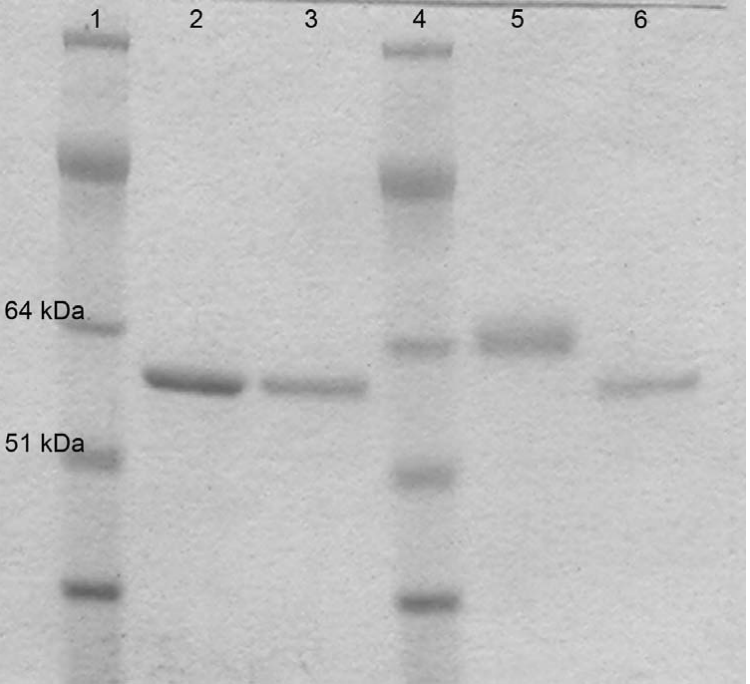
NuPAGE^® ^Bis-Tris SDS-PAGE gel with MOPS buffer, Trichoderma expressed Cel7A in Lane 2 is compared to the higher apparent molecular weight of the untreated Aspergillus expressed rCel7A in Lane 5. An apparent reduction in MW following PNGaseF treatment of rCel7A is demonstrated Lane 6. When treated with PNGaseF the Trichoderma expressed Cel7A does not appear to have a reduced apparent MW as shown in Lane 3. Lanes 1 and 4 contain SeeBlue Plus2 MW standards.

The Cel7A enzymes were deglycosylated using *N*-glycosidase F (PNGaseF) (P0705, New England Biolabs, Inc.) according to manufacturer recommendations. Deglycosylation was conducted both under denaturing and native conditions, depending on the end-use of the deglycosylated enzyme. Using the Glycoprotein Denaturing Buffer (0.5% SDS, 0.04 M DTT, 0.1%) under denaturing conditions (100°C for 10 minutes), approximately 500 U of PNGaseF was used to treat 20 μg denatured Cel7A enzymes. The reactions were incubated overnight at 37°C with shaking and checked by SDS-PAGE. Under non-denaturing conditions, 500 U of PNGaseF was used to treat 100 μg of purified Cel7A without the use of denaturing reagent. The reactions were monitored for 48 h by SDS-PAGE, by the end of which only partial deglycosylation was achieved. On the second day, an additional 500 U of PNGaseF was added and allowed to react overnight. SDS-PAGE analysis on the third day showed that the deglycosylation reaction was near completion shown in Figure 1. The deglycosylated Cel7A enzymes were used directly in the cellulose digestion time course experiments described below.

### N-glycosylation analysis by Mass Spectrometry

Mass spectrometry analyses were performed with a Waters Q-Tof micro quadrupole mass spectrometer (Waters/Micromass, Milford, MA) with an electrospray ionization (ESI) source. HPLC separations were performed using a Waters 2795 HPLC (Waters, Milford, MA). Both HPLC and mass spectrometer are controlled by the Masslynx service pack 4.0 software (Waters/Micromass, Milford, MA). An injection volume of 15 μL of the Cel7A papain digests were separated at 28°C on a C8 column (4.60 mm × 15 cm × 5.0 μm Agilent Zorbax Eclipse XDB-C8) at a flow rate of 100 μL/min. The optimal gradient determined for separation was 90% solvent A (deionized water with 0.2% formic acid) and 10% solvent B (acetonitrile with 0.2% formic acid), linearly changed to 15% A and 85% B after 30 min and 5% A and 95% B after 75 min. Conditions were returned to original conditions of 90% A and 10% B for an additional 10 min to recondition the column before the next injection. The autosampler chamber was held at 4°C throughout the analysis. The separated sample was flowed directly into the mass spectrometer and ionized by positive mode electrospray. The mass spectrometer was tuned with GFP (Waters, Milford, MA) and calibrated with sodium rubidium iodide (Waters, Milford, MA) to cover the mass range examined. The MS data was recorded as survey scans in the range from 400 to 3500 atomic mass units (amu) in positive ES mode. The inlet conditions were set at a capillary voltage of 3000 V, sample cone voltage of 40 V, source temperature of 100°C and a desolvation temperature of 250°C. A desolvation gas flow rate of 650 L/hr flow and a cone gas flow rate of 30 L/hr were used. The collision energy was set at 10 V, which is optimal for steering the electrons to the time-of-flight (TOF) analyzer. The MCP detector and probe positioning was adjusted for optimal sample sensitivity. Run time was set to correspond with the HPLC program that allowed adequate time to separate, collect, and analyze the samples.

### Labeling cellulases with fluorescent tags

The Cel7A enzymes and the CDs were labeled with Alexa Fluor 488 fluorophore (Invitrogen) using the manufacturer recommended labeling protocol. Degrees of labeling (DOL) of 1 to 10 moles dye/mole cellulase were achieved. Fluorescence-labeled cellulases were diluted with unlabeled cellulases to final DOL in the range of 0.5 to 1 for the cellulose hydrolysis assays. The activities of the labeled enzymes on BC and PASC were checked against corresponding unlabeled enzymes, which verified that labeling did not alter the original activity [32].

### Cellulose hydrolysis time course experiments

Reactions containing 1.0 μM Cel7A (intact, CDs, or deglycosylated) and 1.0 mg/mL BC or PASC in 0.25 mL reaction volumes were conducted at 38°C. Triplicate reactions were assayed over the course of 120 h. Each reaction was setup by preparing the appropriate dilution of cellulase in 5 mM sodium acetate buffer (pH 5.0) in 0.5 mL microcentrifuge tubes. The substrate (BC or PASC) was pre-incubated separately at 38°C for a minimum of 30 min. The reactions were initiated by addition of the pre-incubated substrate and incubated at 38°C with end-over-end rotation. At the designated times, the reactions were terminated by separating the liquid and solid phase by filtration in a manifold filtration system equipped with a 96-well 1.0 μM glass fiber filter frit (Innovative Microplate, Chicopee, MA). Reducing sugar concentration was determined by the disodium-2,2'-bicinchoninate (BCA) method [33] using cellobiose for the standard curve. If fluorescence-labeled enzymes were used, the concentration of Cel7A bound to the cellulose fibers was determined by fluorometry as described previously [34]. The solid phase retained in the filter was re-suspended in 250 μL distilled water. Fluorescence intensity of 150 μL of the re-suspended solids was measured in a Tecan GENios fluorometer (Tecan Systems Inc., San Jose, CA) at fluorescence excitation/emission wavelengths of 485 and 535 nm, respectively. Cellulase concentrations on the solids fractions were determined with a set of purified Cel7A standards of known concentrations that was measured concurrently.

## Results

### SDS-PAGE analysis

The rCel7A has a higher apparent molecular weight than Trichoderma expressed Cel7A when analyzed by SDS-PAGE (Figure 1). The rCel7A band also appears broader and more diffuse which is typical of protein with polydispersity of molecular weight due to glycosylation (Figure 1). The amidase *N*-glycosidase F (PNGaseF) specifically cleaves between *N*-linked glycans and the Asn residue of the glycoprotein backbone. Efficacy of PNGaseF requires a minimum *N*-glycan size of GlnNAc_2_Man_3. _PNGaseF treatment of Cel7A did not cause a visible shift in the protein band on SDS-PAGE, indicating a general lack of *N*-linked glycans larger than GlnNAc_2_Man_3 _on the native Cel7A. However, PNGaseF treatment resulted in a significant shift of the rCel7A band to a lower apparent molecular weight, demonstrating the presence of higher order *N*-linked glycans. The deglycosylated rCel7A band appears narrower and sharper than the parent, indicating less mass polydispersity. The mass of the PNGaseF deglycosylated rCel7A is similar to that of Cel7A, providing further evidence that the native Cel7A enzymes are most likely minimally populated at the *N*-linked glycosylation sites.

### Mass spectrometric (MS) analysis

**Figure 2:**
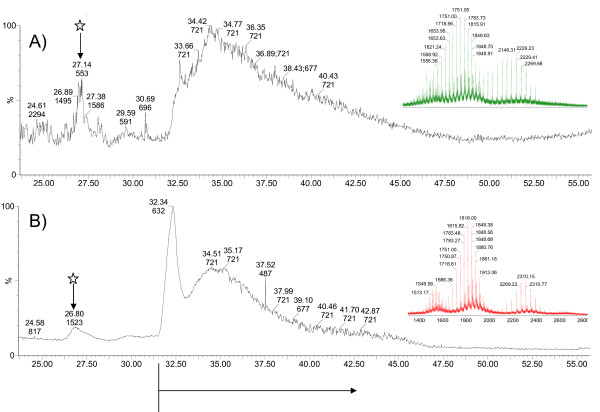
Total ion chromatogram (TIC) of RP-LC elution of papain cleaved A) Cel7A and B) rCel7A. Arrows with a star indicate the peaks containing the linker peptide + CBM fragment. The summed spectra for the linker peptide + CBM peaks shown in insets. Arrow beneath the time axis indicates elution of the CD.

**Figure 3:**
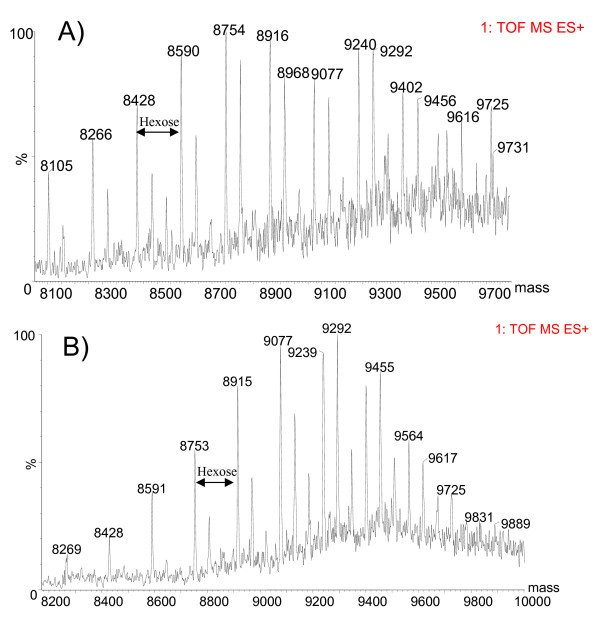
Reconstructed molecular masses of the linker peptide + CBM fragment of A) Cel7A and B) rCelA.

**Figure 4:**
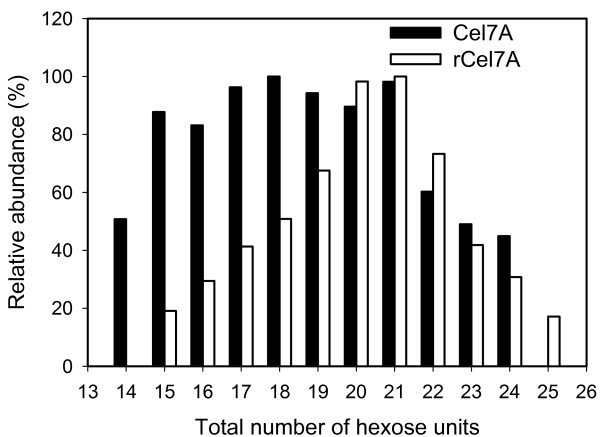
Comparison of the relative abundances of total hexose units on the linker peptide regions of Cel7A and Aspergillus expressed rCel7A.

Papain proteolysis of Cel7A cleaves the protein between two glycine residues (Gly439 and Gly440) at the *C*-terminal end of the catalytic domain (CD), separating the CD from the linker peptide and cellulose binding module (CBM) [31]. The glycosylation patterns on these two fragments were analyzed separately by MS, using this previously demonstrated strategy [19,20]. When separated on a reverse-phase column, the linker + CBM fragment elutes before the CD fragment (Figure 2). The total mass ranges reconstructed from the respective spectra of the linker peptide + CBM fragment from both Cel7A proteins are shown in Figure 3. The dominant masses in Figure 3. are separated by 162 amu, corresponding to the mass of a hexose b-ion. A second series off-set by 57 amu from the dominant series can be observed, most likely attributable to degenerate cleavage by papain on either side of Gly439 [20]. Total hexose counts were estimated from the differences between the observed masses from the spectra and the calculated mass of the non-glycosylated linker peptide + CBM (Gly439 to Leu497) (Figure 4). Although the type of hexose cannot be explicitly determined from the current analysis, hexoses of *O*-glycans on the linker peptide of *T. reesei *Cel7A have been shown to be primarily mannose [20]. As shown in Figure 4, the Cel7A linker contains 14 to 24 total hexoses and rCel7A linker contains 15 to 25 total hexoses. The distribution and abundance of hexoses on the linker regions differ, with rCel7A containing a higher content than Trichoderma expressed Cel7A

**Figure 5:**
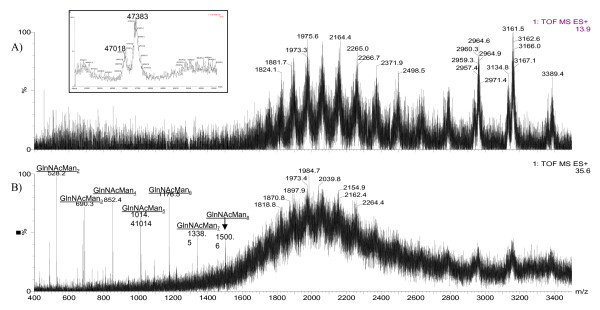
Summed mass spectra of peaks containing CD fragments of A) Cel7A and B) rCel7A. Inset in A) shows reconstructed mass profile of Cel7A CD.

The reconstructed molecular mass profile of the Cel7A CD (Figure 5 inset) shows two prominent average masses at 47.0 and 47.4 kDa. Subtracting the calculated mass of the non-glycosylated CD (Gln1 to Gly439) of 46.4 kDa, yields the total mass contribution of the CD glycans near 1000 Da. There are three known *N*-linked glycosylation sites on the CD (N45, N270 and N384). Therefore, on average, each site is likely populated by one to two *N*-acetylglucosamine (NAG) units (e.g., 203 or 423 Da, respectively). With the mass of the glycosylated linker ranging from 8.1 to 9.7 kDa (Figure 3A), the average mass range of the protein is thus 54.5 to 56.1 kDa, agreeing well with the estimate by SDS-PAGE analysis (Figure 1).

Accurate reconstruction of the mass profile of rCel7A CD was not possible due to the extreme heterogeneity in the masses. Instead, the intact protein was analyzed by MALDI-MS to reveal an average overall molecular weight of 61835 Da, with a 1/2 height peak width of 2400 Da. Subtracting the mass range of the glycosylated linker peptide and CBM (8.2 to 9.9 kDa) (Figure 5B) gives the mass range of the CD as 52.4 to 53.1 kDa. Further subtracting the calculated mass of the non-glycosylated CD results in 6 to 6.7 kDa as the total mass contribution of *N*-linked glycosylation on this CD. The mass spectrum of the rCel7A CD (Figure 5B) prominently features b-ions corresponding to *N*-glycan masses that fragmented at the reducing-end of the first *N*-acetylglucosamine (GlnNAc) unit attached to the protein backbone. Although only up to GlnNAcMan_8 _is highlighted in Figure 5B, the series of *N*-linked glycan masses was observed throughout the detection range (400 to 3500 amu), up to GlnNAcMan_20_. These masses were absent from the mass spectra of Cel7A CD (Figure 5A).

The mass contributions of the glycans on Cel7A and rCel7A determined from the above analyses are summarized in Table 1.

**Table 1:** A summary of mass contributions of the glycans on Cel7A and rCel7A.

Mass Contributions	Cel7A	rCel7A
Protein backbone	52,246 Da	52,246 Da
N-linked glycans on CD	~1 kDa	6 – 6.7 kDa
O-linked glycans on linker peptide	8.1 – 9.7 kDa	8.2 – 9.9 kDa

Total	61 – 63 kDa	66 – 69 kDa

### Activity measurements

**Figure 6:**
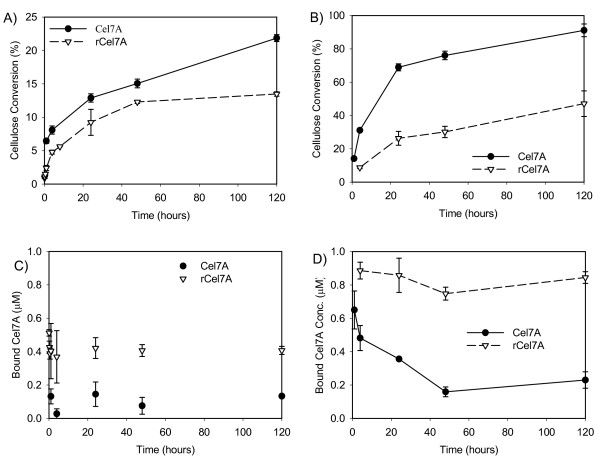
Activities of Cel7A and rCel7A are compared on two cellulose substrates at 38°C. The extent of BC hydrolysis (panel A) is compared to the hydrolysis of PASC (panel B). The respective levels of bound enzyme is shown in panels C and D. Lines are drawn only to guide the eye.

Detailed studies on the purified Cel7A enzymes were conducted on two forms of cellulose, BC and PASC. Comparing Cel7A activities on these two substrates provides a measure of the effect of cellulose recalcitrance on the cellobiohydrolase activities. PASC is a less structured form of cellulose than BC, resulting in faster initial hydrolysis and a further extent of cellulose conversion after five days (Figure 6A,B). Figure 6 shows that Cel7A is more active on both forms of cellulose compared to rCel7A, achieving higher rates and extents of cellulose hydrolysis even with less enzyme bound to the substrate. On PASC, less Cel7A stayed bound compared to rCel7A which remained at 85 to 95% adsorption throughout the reaction (Figure 6D). The corresponding cellulose conversion curves in Figure 6B show that the rates of cellulose conversion by the two forms of Cel7A on PASC in the later phase of the reaction (24 to 120 h) are similar, despite the approximately 4-fold difference in bound concentrations.

**Figure 7:**
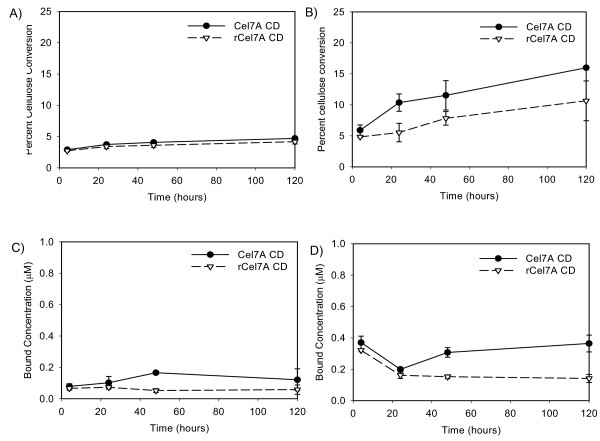
The activities of Cel7A and rCel7A catylitic domains are compared on two cellulose substrates at 38°C. The extents of BC cellulose hydrolysis (panel A) is compared to the hydrolysis of PASC (panel B). The respective levels of bound enzyme are shown in panels C and D.

The cellulose activity experiments were repeated with the CDs of the two enzymes (Figure 7). The activities were considerably reduced when the Cel7A enzymes were devoid of the linker peptides and CBMs. The rate and extent of conversion of BC and PASC by Cel7A CD were lower than that of intact Cel7A, but bound concentrations were comparable for the two enzyme forms (Figure 7C and 7D). This suggests that the access to the substrate did not change upon removal of the linker peptide and CBM, but the activity of the enzyme itself was compromised. The rCel7A CD also showed reduced activity on BC and PASC (Figure 7A and 7B), but had a four-fold reduction in bound enzyme concentration compared to intact rCel7A. When the extent of cellulose hydrolysis by both CDs were compared to the intact enzymes, the same trend held where Cel7A had higher activity than rCel7A (Figure 6A and 7B). However, the differences observed with the CDs were not as pronounced as those observed with the intact Cel7A enzymes.

### Cellobiohydrolase activity of deglycosylated T. reesei Cel7A

Cel7A enzymes that were deglycosylated by PNGaseF under non-denaturing conditions were used to assess the impact of *N*-linked glycans on the cellobiohydrolase activities. Considering the reported specificity for *N*-linked glycans, the PNGaseF treated Cel7A enzymes should retain all *O*-linked glycans. The *N*-deglycosylated Cel7A enzymes were incubated with BC and PASC for 24 h and the hydrolysis results are shown in Table 2. Control reactions using Cel7A and rCel7A on BC were run simultaneously, yielding conversions of 11.9% (± 0.06) and 9.7% (± 0.3), respectively, agreeing with the conversions using the fluorescence-labeled Cel7As (Figure 6, Table 2). The extents of cellulose hydrolysis by the deglycosylated Cel7As cannot be directly compared to those of the untreated Cel7As as the data suggests that the deglycosylation protocol may have compromised enzyme activity. One reason may be that because PNGaseF was not removed, its presence in the reaction mix may interfere with Cel7A activity.

**Table 2:** Comparing extents of cellulose hydrolysis after 24 hours at 38°C.

		On BC		On PASC	
		
Enzyme	Modification	^1^Cellulose Hydrolysis (%)	Std. dev.	^1^Cellulose Hydrolysis (%)	Std. dev.
Cel7A	None	12.9	0.6	69.0	2.1
	^3^CD-only	3.7	0.2	10.4	1.4
	^4^PNGaseF treated	^#^5.6	0.2	^#^31.4	1.6

rCel7A	None	9.2	2.0	26.3	4.1
	^3^CD-only	3.4	0.3	5.5	1.5
	^4^PNGaseF treated	^#^5.1	0.1	^#^26.9	0.5

^1^Data for the intact and CD forms are also plotted in Figure 6 and Figure 7.

^3^Purified from papain digests (see Materials and Methods).

^4^Deglycosylated by PNGaseF under native conditions (see Materials and Methods).

^#^Activity of deglycosylated enzyme may have been compromised by the deglycosylation protocol and cannot be directly compared to the activity of the intact enzyme.

**Figure 8:**
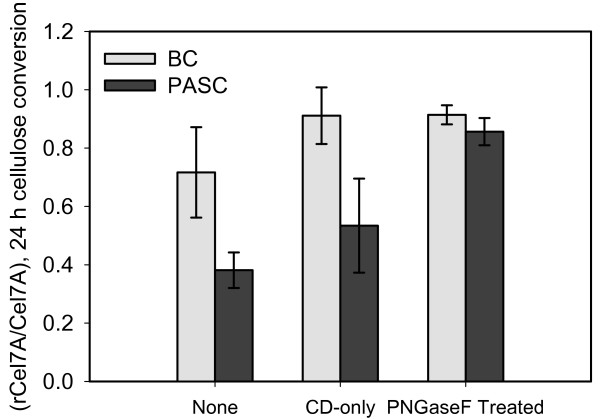
The ratios of BC or PASC hydrolysis in 24 h by rCel7A to that by Cel7A. Standard deviations for the ratios were calculated from measured values (Table 2), assuming no covariance between cellulose hydrolysis by Cel7A and rCel7A.

Comparing the activities of the various forms of rCel7A to Cel7A at 24 h does yield some interesting observations. Figure 8 shows ratios of the extents of BC or PASC hydrolysis at 24 h by various forms of rCel7A to the corresponding forms of Cel7A. In this figure, a ratio of 1.0 would indicate that rCel7A and Cel7A had similar activities, and ratios of lower than 1.0 would indicate that the activity of rCel7A was lower than that of Cel7A.

On PASC, the ratio of hydrolysis at 24 h by rCel7A to Cel7A was 0.38 ± 0.06. The hydrolysis ratio did not improve significantly (0.53 ± 0.16) when only the CDs were reacted with PASC. Deglycosylated rCel7A, however, hydrolyzed 86% as much PASC as did the Cel7A (ratio of 0.86 ± 0.05). These results demonstrate that although rCel7A is only half as active as Cel7A on PASC, PNGaseF treatment of rCel7A restores a significant portion of the native activity. Although the data on BC is confounded by the large magnitudes of the standard deviations, there is still a significant improvement in the relative activity of rCel7A on BC due to the PNGaseF treatment.

## Discussion

We found that binding affinity of Cel7A on cellulose was reduced without its linker peptide and CBM at the early stages of digestion. Generally it is felt that the CBM is responsible for the adsorption of cellulases, such as Cel7A to cellulose. The observed rates and extents of hydrolysis were lower for the CD than for the intact Cel7A, suggesting the specific activity (i.e. turnover rate) of the bound enzyme was significantly reduced by the absence of the linker and CBM. It is also important to note that BC prepared as described here has a reported content of microcrystalline cellulose of about 80–85% [35], which means that the digestion observed here for the CDs (~5% at 120 h) does not represent conversion comparable with that found for the intact enzymes (~25% at 120 h); i.e., the CD conversion is probably due to hydrolysis of amorphous cellulose content in BC. These data support the concept that the linker peptide and CBM are essential for optimum activity of Cel7A. The Cel7A enzyme is thought to act processively from the reducing ends of cellulose (21). The linker peptide and CBM may; therefore, be important for proper processive action of the enzyme.

Heterologous expression of *T. reesei *Cel7A in *A. niger var. awamori *compromised its activity on cellulose. Deglycosylation of the rCel7A using PNGaseF demonstrates that *N*-linked glycan can reduce the specific activity of the enzyme. The higher *N*-glycan content on rCel7A appeared to be at least partially responsible for this reduction, with the potential for recovering partial native activity by enzymatic deglycosylation. The rCel7A showed higher binding on cellulose but conversion was lower than the native enzyme, suggesting that rCel7A may be more prone to non-productive binding to cellulose. In contrast to Cel7A, the affinity of rCel7A to cellulose was reduced upon elimination of the linker peptide and CBM. It seems thus that the linker peptide and CBM promotes non-productive binding of rCel7A to cellulose. One possible explanation is that the larger *N*-linked glycan structures on the CD of rCel7A interact with the linker peptide and/or CBM to increase the likelihood of non-productive binding. Another possibility is that the *N*-linked glycans of rCel7A may interact with the cellulose surface to interfere with the binding and function of the enzyme

Contrary to the activity differences observed with purified Cel7As, previous comparisons of Cel7A and rCel7A in binary saccharification assays with *Acidothermus cellulolyticus *E1 endoglucanase showed very similar activities on pretreated corn stover (PCS) (1). The contrasts observed with purified monocomponent digestion; therefore, potentially highlight mechanistic differences that can perhaps be overcome in a synergistic mixture, especially where the mixtures generate much higher degrees of conversion of microcrystalline cellulose (i.e., >20% conversion). The binary enzyme saccharification assays on pretreated yellow poplar (PYP); however, showed decreased activity when using rCel7A compared to the native (1), suggesting that substrate properties can also be a strong influencing factor. Synergistic cellulase activities (such as the presence of endoglucanases) may overcome some of the differences in substrate properties. For example, if increased glycosylation interferes with the processivity of rCel7A, causing the enzyme to disengage from the substrate more frequently, and then having sufficient endoglucanase-created reducing-ends could mitigate the effects of this handicap.

## Conclusion

In this study, we demonstrated that fungal heterologous expression of *T. reesei *Cel7A could affect the final *N*-linked glycosylation of the protein and result in compromised activity on cellulose. If it is necessary to utilize a non-native expression host for *T. reesei *cellulases, *in vitro *processing of the protein glycosylation may be required to maximize the activity of these enzymes.

## Competing interests

The authors declare that they have no competing interests.

## References

[B1] Perlack RD, Wright LL, Turhollow AF, Graham RL, Stokes BJ, Erbach DC, Agriculture USD, Energy USD (2005). Biomass as feedstock for a bioenergy and bioproducts industry:  The technical feasibility of a billion-ton annual supply.

[B2] Coutinho PM, Henrissat B, Gilbert HJ, Davies G, Henrissat B, Svensson B (1999). Carbohydrate-active enzymes:  an integrated database approach. Recent Advances in Carbohydrate Bioengineering.

[B3] Zhang YHP, Himmel ME, Mielenz JR (2006). Outlook for cellulase improvement: Screening and selection strategies. Biotechnology Advances.

[B4] Nevalainen KMH, Te'o VSJ, Bergquist PL (2005). Heterologous protein expression in filamentous fungi. Trends in Biotechnology.

[B5] Imperiali B, O'Connor SE (1999). Effect of N-linked glycosylation on glycopeptide and glycoprotein structure. Current Opinion in Chemical Biology.

[B6] Godbole S, Decker SR, Nieves RA, Adney WS, Vinzant TB, Baker JO, Thomas SR, Himmel ME (1999). Cloning and expression of Trichoderma reesei cellobiohydrolase I in Pichia pastoris. Biotechnology Progress.

[B7] Boer H, Teeri TT, Koivula A (2000). Characterization of Trichoderma reesei cellobiohydrolase CeI7A secreted from Pichia pastoris using two different promoters. Biotechnol Bioeng.

[B8] Den Haan R, Rose SH, Lynd LR, van Zyl WH (2007). Hydrolysis and fermentation of amorphous cellulose by recombinant Saccharomyces cerevisiae. Metabolic Engineering.

[B9] Penttila ME, Andre L, Lehtovaara P, Bailey M, Teeri TT, Knowles JK (1988). Efficient secretion of two fungal cellobiohydrolases by Saccharomyces cerevisiae. Gene.

[B10] Foreman PK, Brown D, Dankmeyer L, Dean R, Diener S, Dunn-Coleman NS, Goedegebuur F, Houfek TD, England GJ, Kelley AS, Meerman HJ, Mitchell T, Mitchinson C, Olivares HA, Teunissen PJM, Yao J, Ward M (2003). Transcriptional regulation of biomass-degrading enzymes in the filamentous fungus Trichoderma reesei. Journal of Biological Chemistry.

[B11] Henrissat B, Driguez H, Viet C, Schulein M (1985). Synergism of cellulases from Trichoderma reesei in the degradation of cellulose. Bio-Technology.

[B12] Nummi M, Niku-Paavola ML, Lappalainen A, Enari TM, Raunio V (1983). Cellobiohydrolase from Trichoderma reesei. Biochemical Journal.

[B13] Barr BK, Hsieh YL, Ganem B, Wilson DB (1996). Identification of two functionally different classes of exocellulases. Biochemistry.

[B14] Kipper K, Valjamae P, Johansson G (2005). Processive action of cellobiohydrolase Cel7A from Trichoderma reesei is revealed as 'burst' kinetics on fluorescent polymeric model substrates. Biochemical Journal.

[B15] Palonen H, Tjerneld F, Zacchi G, Tenkanen M (2004). Adsorption of Trichoderma reesei CBH I and EG II and their catalytic domains on steam pretreated softwood and isolated lignin. Journal of Biotechnology.

[B16] Palonen H, Tenkanen M, Linder M (1999). Dynamic interaction of Trichoderma reesei cellobiohydrolases Cel6A and Cel7A and cellulose at equilibrium and during hydrolysis. Appl Environ Microbiol.

[B17] Zhang YH, Lynd LR (2006). A functionally based model for hydrolysis of cellulose by fungal cellulase. Biotechnol Bioeng.

[B18] Stals I, Sandra K, Geysens S, Contreras R, Van Beeumen J, Claeyssens M (2004). Factors influencing glycosylation of Trichoderma reesei cellulases. I: Postsecretorial changes of the O- and N-glycosylation pattern of Cel7A. Glycobiology.

[B19] Hui JPM, White TC, Thibault P (2002). Identification of glycan structure and glycosylation sites in cellobiohydrolase II and endoglucanases I and II from Trichoderma reesei. Glycobiology.

[B20] Harrison MJ, Nouwens AS, Jardine DR, Zachara NE, Gooley AA, Nevalainen H, Packer NH (1998). Modified glycosylation of cellobiohydrolase I from a high cellulase-producing mutant strain of Trichoderma reesei. European Journal of Biochemistry.

[B21] Eriksson T, Stals I, Collen A, Tjerneld F, Claeyssens M, Stalbrand H, Brumer H (2004). Heterogeneity of homologously expressed Hypocrea jecorina (Trichoderma reesei) Cel7B catalytic module. European Journal of Biochemistry.

[B22] Garcia R, Cremata JA, Quintero O, Montesino R, Benkestock K, Stahlberg J (2001). Characterization of protein glycoforms with N-linked neutral and phosphorylated oligosaccharides: studies on the glycosylation of endoglucanase 1 (Cel7B) from Trichoderma reesei. Biotechnol Appl Biochem.

[B23] Maras M, DeBruyn A, Schraml J, Herdewijn P, Claeyssens M, Fiers W, Contreras R (1997). Structural characterization of N-linked oligosaccharides from cellobiohydrolase I secreted by the filamentous fungus Trichoderma reesei RUTC 30. European Journal of Biochemistry.

[B24] Eneyskaya EV, Kulminskaya AA, Savel'ev AN, Shabalin KA, Golubev AM, Neustroev KN (1998). alpha-mannosidase from Trichoderma reesei participates in the postsecretory deglycosylation of glycoproteins. Biochem Biophys Res Commun.

[B25] Maras M, Callewaert N, Piens K, Claeyssens M, Martinet W, Dewaele S, Contreras H, Dewerte I, Penttila M, Contreras R (2000). Molecular cloning and enzymatic characterization of a Trichoderma reesei 1,2-alpha-D-mannosidase. Journal of Biotechnology.

[B26] Adney WS, Chou YC, Decker SR, Ding SY, Baker JO, Kunkel G, Vinzant TB, Himmel ME (2003). Heterologous expression of Trichoderma reesei 1,4-beta-D-glucan cellobiohydrolase (Cel 7A). Applications of Enzymes to Lignocellulosics.

[B27] Hestrin S, Schram M (1954). Synthesis of cellulose by Acetobacter xylinum. Biochemical Journal.

[B28] Krystynowicz A, Czaja W, Wiktorowska-Jezierska A, Goncalves-Miskiewicz M, Turkiewicz M, Bielecki S (2002). Factors affecting the yield and properties of bacterial cellulose. Journal of Industrial Microbiology & Biotechnology.

[B29] Helbert W, Chanzy H, Husum TL, Schulein M, Ernst S (2003). Fluorescent cellulose microfibrils as substrate for the detection of cellulase activity. Biomacromolecules.

[B30] Sangseethong K, Penner MH (1998). p-Aminophenyl beta-cellobioside as an affinity ligand for exo-type cellulases. Carbohydrate Research.

[B31] Vantilbeurgh H, Tomme P, Claeyssens M, Bhikhabhai R, Pettersson G (1986). Limited proteolysis of the cellobiohydrolase I from Trichoderma reesei - separation of functional domains. Febs Letters.

[B32] Jeoh T, Ishizawa CI, Davis MF, Himmel ME, Adney WS, Johnson DK (2007). Cellulase digestibility of pretreated biomass is limited by cellulose accessibility. Biotechnology and Bioengineering.

[B33] Doner LW, Irwin PL (1992). Assay of reducing end-groups in oligosaccharide homologs with 2,2'-bichinchoninate. Analytical Biochemistry.

[B34] Jeoh T, Wilson DB, Walker LP (2002). Cooperative and competitive binding in synergistic mixtures of Thermobifida fusca cellulases Ce15A, Ce16B, and Ce19A. Biotechnology Progress.

[B35] Udhardt U, Hesse S, Klemm D (2005). Analytical investigations of bacterial cellulose.. Macromolecular Symposia.

